# New Model for Analytical Predictions on the Bending Capacity of Concrete Elements Reinforced with FRP Bars

**DOI:** 10.3390/ma14030693

**Published:** 2021-02-02

**Authors:** Kostiantyn Protchenko, Przemysław Leśniak, Elżbieta Szmigiera, Marek Urbański

**Affiliations:** Department of Civil Engineering, 16 Armii Ludowej Av., Warsaw University of Technology, 00-637 Warsaw, Poland; przemyslaw.lesniak.stud@pw.edu.pl (P.L.); e.szmigiera@il.pw.edu.pl (E.S.); m.urbanski@il.pw.edu.pl (M.U.)

**Keywords:** fibre reinforced polymers FRP, FRP bars, FRP reinforced concrete members, statistical analyses, Nonlinear Generalized Reduced Gradient (GRG) optimization

## Abstract

Many studies on Fibre-Reinforced Polymers Reinforced Concrete (FRP-RC) beams tested in flexure have been performed by various researchers around the world. This work presents the results of statistical and mathematical analyses based on experimental data; 102 samples were collected and supplemented from 16 different scientific papers. The load capacity of the beams determined on the basis of the tests was compared with the load capacity calculated on the basis of the recommendations of ACI 440.1R-15. The results obtained from experimental studies showed that for 91.4% of the samples, the underestimation of the load capacity on average was equal to 15.2% of theoretical, and for 33.3% of the beams, the load capacity was overestimated by 26.7%. The paper proposes a new empirical coefficient incorporating material parameters to be implemented into ACI 440.1R-15 flexural design approach in order to improve the accuracy of this model in scope of the nominal flexural strength capacity of FRP-reinforced beams estimation. Modifications to flexural design of FRP-RC beams with the use of ACI 440.1R-15 design code were proposed. As a result, the reliability of the analytical model is increased; therefore, the new model guarantees higher safety and cost efficiency of designed concrete structures reinforced with FRP bars.

## 1. Introduction

Design of concrete members reinforced with fibre-reinforced polymers (FRP) bars is based on the design of concrete members reinforced with ordinary steel bars. However, the behaviour of these two materials is significantly different; therefore, to ensure safety and efficiency of FRP reinforcement, a deep understanding of the nature of this material and the adequate use of available design codes is required.

An important issue related to FRP bars is their deformation due to creep, which may eventually lead to failure of FRP bar due to creep rupture. The creep effect is initially related to polymer matrix when it occurs; further, this stress is transferred substantially to enhancing fibres. When enhancing fibre is broken, its internal stresses have to be transferred by outer fibres. Very often, such an event works as a chain effect, causing the entire FRP bar to fracture. There are many scientific materials available regarding failure mechanisms of FRP bars since this is one of the key issues in the use of this material as internal reinforcement in concrete structures. Numerous experimental research studies prove that the character of failure of FRP bars is very complex and dependent on a large number of varying factors. It has been observed in tensile tests of FRP bars that various types of FRP bars fracture in slightly different ways.

Although higher efficiency of bars is achieved with a smaller diameter (higher strength with relation to cross-section area) due to the shear lag effect, FRP bars with larger diameter fracture in a more gradual manner. Failure of FRP bars with a small diameter has a relatively more sudden character [1].

Steel bars are characterised by generalised values of mechanical properties, which are widely standardized. FRP reinforcement is available in the market in various types and subtypes with a wide range of properties. Material characteristics of FRP bars are guaranteed by manufacturers and usually represent initial values, which undergo (often difficult-to-predict) changes with time and exposure to certain environmental conditions. In the design of reinforced concrete members, these potential degradations of mechanical properties are covered with the use of environmental reduction factorswhich takes into account environmental effects and type of FRP bars [2,3,4].

In accordance with FIB bulletin 14, the design strength can be obtained by dividing the characteristic strength by a partial safety factor. The partial safety factors applied to the characteristic strength of FRP are mainly based on the observed differences in the long-term behaviour of FRP as well as the application method and on-site working conditions [5].

The design codes and recommendations regarding FRP reinforcement are mainly based on the semiprobabilistic method of limit states. Among the available recommendations addressing the subject of FRP reinforcement can be distinguished a Japanese recommendation JSCE [6], a set of Canadian recommendations for cubature buildings CSA-S806-12 (2012) [7], and, for bridge structures, CHDBC (2006) [8], a set of American recommendations ACI 440.1R-06 (2006) [9] and ACI 440.1R-15 (2015) [4] and a set of Italian recommendations CNR-DT 203/2006 (2006) [10].

The JSCE recommendations define the safety factors related to the FRP-RC members and to the constituent material. The CSA recommendations provide the characteristics of testing strength properties of FRP bars. Recommendations ACI 440 introduces material reduction factors for elements subjected to bending in the ultimate limit state and reduction factors corresponding to the failure mechanism of FRP RC member. Recommendations addressing the subject of FRP reinforcement are based, among other things, on analytical solutions and empirical equations resulting from research carried out on samples of FRP bars and concrete elements reinforced with FRP bars. Due to the different mechanical properties of FRP bars, the recommendations introduce modifications to the coefficients available in existing standards concerning concrete elements reinforced with steel bars.

The purpose of strength reduction factors proposed in 440.1R-15 design code is to assure flexural strength reserve of FRP-reinforced beams included in the design capacity of FRP reinforced beam, i.e., in nominal calculated factored bending moment capacity. According to ACI 440 analytical model and considering experimental data from other studies, design flexural strength of FRP reinforced beam with a compression-controlled cross-section is estimated to be 18% higher than for FRP-reinforced beam with a tension-controlled cross-section. Strength reduction factors are applied to values of nominal flexural strength of concrete beam reinforced with FRP, which in most cases (91.4% of data used for research in this work, i.e., over 9 of 10 considered specimens in are underestimated. Of ACI 440.1R-15 theoretical results, 56.2% are underestimated by 10% or more, and 26.7% of ACI 440.1R-15 theoretical results are underestimated by 20% or more. The highest underestimations reached 39%.

This work discusses and analyses factors responsible for such inaccuracies in theoretical results, proposes a solution to this issue in form of proposed modifications, and introduces a new coefficient into ACI 440.1R-15 flexural design approach. The purpose of implementation of new coefficient into ACI 440.1R-15 analytical model is to increase the accuracy of this model in the scope of the nominal flexural strength capacity of FRP reinforced beams estimation. Additional parameters in ACI 440.1R-15 formulas significantly increases the accuracy of theoretical results. Proposed modifications concern the use of Glass FRP (GFRP) and Carbon FRP (CFRP) bars as reinforcement and consider the influence of defined factors related to concrete beams on material characteristics of these bars.

Before proceeding to statistical analysis, the conditionality related to examined specimens was determined and analysed. Necessary considerations, as well as differences and similarities regarding all beams included in current research, are listed in the following parts of the paper.

### Theoretical Background

The load capacity of bending concrete elements with FRP reinforcement is determined using methods analogous to the method of determining the load capacity of bending steel-reinforced concrete elements. The load-bearing capacity, unlike for steel-reinforced concrete elements, is influenced by several important factors mainly based on the characteristics of FRP composite reinforcement. The adhesion of composite bars to concrete has a great influence on the load capacity. There are significant differences between the traditional steel reinforcement and the composite reinforcement in the form of FRP bars. The modulus of elasticity of FRP bars is generally lower than that of steel bars, especially in the transverse direction. The shear stiffness of FRP bars is much lower than that of steel. The surface deformation of FRP bars relates to the resin matrix, which has much lower strength than steel bars. At limit loads (when the load capacity is lost), the importance of adhesion and friction between the bars and the surrounding concrete surface decreases. On the other hand, the wedging effect begins to play an important role, as it increases with the increase of concrete compressive strength. The wedging effect in the case of an appropriate braid angle of the FRP reinforcement influences the bending resistance. On the other hand, due to the lack of plastic deformation of FRP reinforcement, a coefficient related to the degree of reinforcement should be used in the bending sections. The value the ψ factor is related to the change in the degree of reinforcement, which directly affects the failure mode of the concrete bending elements. After deep analyses of the static database of beams, due to the two above-mentioned factors, the authors proposed the coefficient ψ as a function of concrete strength and reinforcement ratio; assuming the ratio of variable to constant loads from 1 to 3 and for the reliability index β in the range from 3.5 to 4.0 (analogous to those shown in ACI 440.1R), it increases the accuracy of the theoretical results. For concrete with compressive strength up to 55 MPa, the reduction factor of the load capacity ranges from 1 to 1.15 with a transition zone depending on the failure mode. For concrete with compressive strength above 55 Mpa, two values of the load capacity reduction factor without transition zone are proposed.

## 2. Research Programme

For experimental testing, a standard four-point loading scheme was applied in all cases. Ratios of middle span length to edge span lengths, as well as reinforcement detailing, did not differ significantly. The dataset used for this research, consisting of 102 positions, included five beams, i.e., 4.9% of all specimens, with square cross-section (152 mm × 152 mm—beams with the smallest cross-section in the dataset); 14 [11,12] stocky beams (b > h), i.e., 13.7%; 44 beams, i.e., 43.1%, with cross-section of 200 mm width and 300 mm depth; 2 specimens, i.e., 2.0%, with350 mm depth; and 2 specimens, i.e., 2.0%, with 550 mm depth with the same width. Dimensions of 35 specimens, i.e., 34.3%, ranged (b × h) from 130 mm × 180 mm to 180 mm × 300 mm. Excluding squared and stocky beams, the ratio of depth to width ranged from 1.36 to 1.50 for 95% of beams—3.9% of all beams with depth higher or equal to 350 mm is characterised by substantially higher ratio.

Beam (span) lengths of FRP concrete beams reinforced with FRP bars were analysed and compared due to the economies of scale. The predominant part (82%) of examined specimens were characterised by span length ranging from 1.5 to 2.75 m considering simple supported beams, as well as continuous beams, and span length of 18% of beams reached up to 3.4 m. The slenderness of beams and the relations between the area of cross-section, dimensions of cross-section, and span length of beams were analysed—obtained data were normally distributed—results significantly deviating from average were not found. On the basis of the analysis of geometrical characteristics of FRP-reinforced beams, the possibility of scale effect occurrence was excluded.

Various methods of application of load to the beams were used. Thériault, M., and Benmokrane, B. [13] tested FRP-reinforced beams under static loading conditions; specimens were subjected to several (from one to four) loading/unloading cycles for additional crack width investigation. Kassem, C., Farghaly, A., and Benmokrane, B. [14] applied load to the beams at a stroke-controlled rate; the loading rate was adjusted to the deflection of the tested specimen. Beams tested by Ashour, A. F. [15] were subjected to load applied in increments of 2 kN, after each increment load was kept constant. Yost, J. R., Goodspeed C. H., and Schmeckpeper, E. R. [11] used a testing machine with a programmed, time-dependent loading rate of 4.45 kN/min. Methods used by other researchers in experimental testing of specimens were analogous to the methods presented above. Despite technical differences in the application of loading (smoothly or gradually), one facet converges for all tested beams: the loading was not applied rapidly but with a low increment.

On the basis of insightful qualitative analysis of conditionality of examined concrete beams reinforced with FRP bars, no differences were found that could significantly affect the interpretation of data and results.

**Figure 1:**
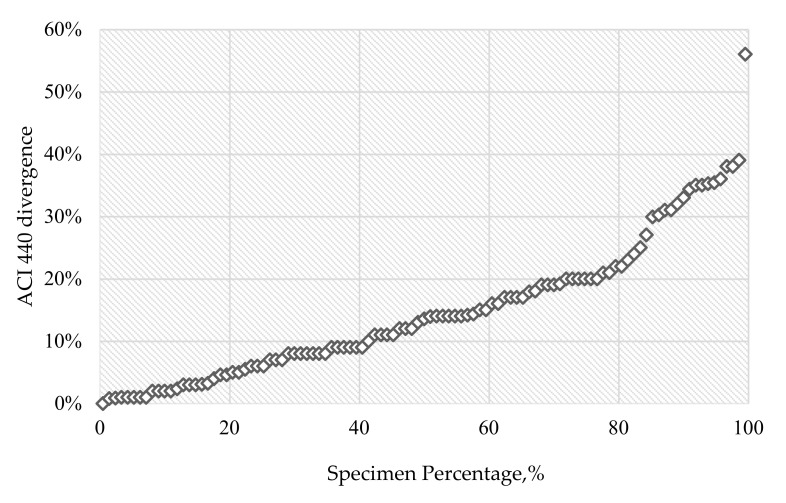
Normal probability plot—original ACI 440 results.

For 32 beams reinforced with CFRP bars, in 2 cases (6.3%), the load capacity was significantly overestimated, while in 4 cases (12.5%), it was significantly underestimated. In the case of 70 beams reinforced with GFRP bars, in 3 cases (4.3%) the load capacity was significantly overestimated, and in 13 cases (18.6%), it was significantly underestimated (compared to theoretical calculations). More detailed (or general) conclusions can be interpreted using Figure 1, where discrepancies (in absolute value) between the theoretical and experimental load bearing are marked. Specimens with significantly overestimated or underestimated flexural strength were analysed individually, incorporating an examination of available data and additional parameters related to these beams, provided in research papers; reasons were not found for any beam.

Due to the small number of beams for which significant and unexplainable (in scope of the data analysed in the current research) deviation occurred, there is the possibility of a significant contribution of factors related to improper specimen preparation or other, e.g., quality-control-related, neglection. For the above reasons, it may be feasible for some positions in the collected data used for statistical and mathematical analysis to be dismissed.

The research programme was structured in a hierarchical manner and is discussed in Table 1. To proceed to the next research task, selected data from the previous one were required. Discussion regarding each research task is included in the appropriate section.

### 2.1. Data on Experimental Flexural Testing of FRP-Reinforced Beams

Flexural design of concrete members reinforced with FRP bars can be based on the same assumptions as the flexural design of concrete members reinforced with steel bars. When designing a beam with conventional steel reinforcement, it is assumed that the reinforcement in the tension zone becomes plasticized, and the concrete is crushed in the compression zone at the same time. Then, the beam cross-section is defined as optimally reinforced. The cross-section of the beam is defined as under-reinforced when the amount of reinforcement is less than necessary for the situation described above. However, if the amount of reinforcement applied is greater, the beam cross-section is called a reinforced cross-section.

Unlike steel reinforcement, there is no plastic deformation phase in tension in the FRP bar reinforcement. The stress-strain relationship in axial stretching in FRP rebars is linearly elastic until failure.

The purely linear elastic nature of this relationship significantly influences the change of the design approach used to determine the load-bearing capacity of concrete elements reinforced with steel bars. The lack of a phase of plastic deformation of FRP bars implies the necessity to change the load capacity design algorithm. In general, the design of steel-reinforced beams avoids the occurrence of concrete crush failure. In the design of FRP beams, this failure mode is preferred because the tied concrete of the beam exhibits a limited level of strength after failure, albeit with a reduced stress level. The method of determining internal forces in reinforced FRP sections is similar to the method used for beams with conventional reinforcement; however, the analysis of the ultimate moment of load capacity of the section is based on taking into account the linearly elastic behaviour of FRP bars.

According to ACI 440.1R-15, design flexural strength in a cross-section of concrete member reinforced with FRP bars should not exceed the factored bending moment. The factored bending moment is a function that results from the geometry of the member, location of FRP bars, and mechanical properties of concrete and FRP bars. The strength reduction factor is associated with the failure mode of the member under flexure and characteristics of materials.

Nominal flexural strength of concrete member reinforced with FRP bars can be computed on the basis of compatibility of strain, an equilibrium of internal forces and equilibrium of strength limit state, i.e., failure modes.

Table 2 includes data from 16 different works of research teams or individual researchers, ranging from 1993 to 2017, and is structured by references. The beams were subjected to flexural testing and were destroyed either due to concrete crushing or reinforcement failure. Data not provided in some research documents, e.g., ACI 440.1R-15 theoretical moment capacities of beams, is elaborated and complemented by information provided by researchers in their works. Experimental results are compared to theoretical results as a ratio of ACI 440 theoretical moment to the result obtained experimentally. The last column in Table 2 is the ratio of load-bearing capacity obtained experimentally to the theoretical value calculated in accordance with ACI. Ratios of experimental-to-theoretical load-bearing for these specimens are marked with bold font in Table 2 for facilitated identification; ratio values outside the range of 0.8–1.0 are shown in bold.

### 2.2. Probability Analysis—Dataset Verification

Results of probability analysis are represented in Figure 1 using a normal probability plot, a technique of statistical data visualisation intended for assessing the distribution of a dataset. All values of divergence between load bearing calculated in accordance with the ACI 440 analytical result and the load bearing obtained by testing corresponding to concrete beams reinforced with FRP bars are plotted with relation to theoretical normal distribution.

The discrepancy (in absolute value) between the theoretical and test load bearing is marked on the vertical axis. On the horizontal axis, the percentage fractions of beams with the corresponding load bearing divergence are given.

Normally distributed data should be visualised on the normal probability plot as a straight line. Deviations from this line are equivalent to divergences from normality [27]. From the plot related to FRP reinforced beams, it can be visually verified that ACI 440 departures are normally distributed up to 82% of all specimens, then from 82% to 99% of all specimens, departure from normality can be observed. One point on the graph departs from normality, an error corresponding to specimen no. 71, (beam CS1b in Table 2).

The change of the slope of the linear fit line at 82 specimen per cent is addressed to ACI 440.1R-15, in opposition to the influence of FRP reinforcement ratio and the influence of superposition of high reinforcement ratio and high compressive strength of concrete on the flexural strength of FRP-reinforced beam. This statement cannot be concluded directly from the normal probability plot; at this stage, it is an explanation of the nature of the normal probability plot, which results from statistical and mathematical analysis.

Results of probability analysis indicate that the dataset is appropriate for further statistical analysis due to the statistical significance parameter, the compressive strength of concrete, and the degree of reinforcement and the combination of these factors, which meets the significance criterion <160 × 10^−6^.

### 2.3. Regression and ANOVA Analysis (Analysis of Variance)

Regression and ANOVA analysis were performed to investigate the influence of eight variables: 1. static scheme; 2. FRP bar type; 3. concrete strength; 4. reinforcement ratio; 5. failure mode; 6. year of research—based on the theoretical ACI 440 deviation of nominal moment capacity of FRP-reinforced beams in flexural design and the superposition of simultaneous occurrence of two significant variables from the statistical point of view—7. concrete strength with reinforcement ratio; and 8. failure mode with reinforcement ratio, which was investigated to verify the existence of an enhanced interference effect. Statistical analysis was performed on the whole dataset, which includes data on all FRP-reinforced beams listed in Table 2.

The main conclusions and results of regression and ANOVA analysis are summarised in Table 3. The variables of concrete strength, reinforcement ratio, and their superposition were used for correlation analysis, to verify which values of these parameters converges with regression analysis set, and further for mathematical analysis, to implement results into an analytical model by effectively adjusting coefficients related to these parameters. Other investigated variables were rejected from the statistical point of view due to the low correlation coefficient or due to logical insignificance, i.e., correlation not corresponding to causation. The influence of variable reinforcement ratio on discrepancies between ACI 440.1R-15 and experimental results was caused by a convergence of high reinforcement ratio values with failure mode concrete crushing. A higher correlation regarding variables containing variable reinforcement ratio over correlation regarding variables containing variable failure mode indicates a variable reinforcement ratio to be a pivotal factor; therefore, parameters 5 and 8 are rejected despite the statistical significance. The variables with significance <160 × 10^−6^ and correlation >0.36 were considered appropriate to use. Such significance indicates nearly zero slope of the linear fit line on linear regression plot; the chance that the result was obtained by chance is nearly zero. Corresponding correlation higher than 36% indicates a high possibility of occurrence of positive or negative correlation at certain parts of the dataset.

Therefore, the criteria for accepting or rejecting the studied variables are the degree of correlation (R squared) >0.36 and significance <160 × 10^−6^. It is clear that with such constraints, out of the eight variable factors presented, only the concrete strength, the degree of reinforcement, and their combination meet the above criteria (the degree of correlation is highlighted by bold).

On the basis of the obtained results, three variables, which are presented below, were selected for further study. Regression analysis results related to three variables, which are proposed to be implemented into the analytical model, are summarised in Table 4, and results of ANOVA analysis are included in Table 5.

Regression analysis for variables included in Table 4 was performed on a complete dataset containing all positions included in Table 2; therefore, the number of observations for each variable is equal to 102 and the corresponding degree of freedom (df in Table 5) is equal to 104. Position R Square in Table 4 is a measure of the percentage of specimens in the dataset, for which discrepancy between analytical and experimental flexural strength of beam can be explained by the influence of specific variable. Values of R Square in Table 4 ranging from 17.4% to 22.6%, and not exceeding 1.7% for excluded variables, in the scope of current research are barely sufficient to be applicable for the dataset containing all specimens. However, on the basis of these results, it can be concluded that the specified part of dataset, eventually consisting of positions containing common qualities or characteristics, may be responsible for highly suggestive values of R Square. This is verified and confirmed with the use of more advanced correlation analysis tools in further work.

Slightly lower values of Adjusted R Square than values of R Square, as well as relatively low values of Standard Error in Table 4 signify that the model (in terms of the number of positions in dataset and diversity of data) used for regression analysis is highly adequate. The very high significance of output of regression analysis is confirmed by nearly zero values of Significance F in Table 5. Components required for computation of significance of regression analysis results—SS (Sum of Squares), MS (Mean Square), and F (F-statistic)—are included in Table 5.

#### 2.3.1. Concrete Strength

According to the results (as given in Table 4 for variable concrete strength), there exists a positive correlation, equal to 41.7%, between concrete strength and ACI 440.1R-15 discrepancy, noted as Multiple R. 17.4% of the variation in the theoretical error of nominal flexural moment predicted with the use of ACI 440.1R-15 design code can be explained by the variation in concrete strength of FRP-reinforced beams a value of R Square related to variable concrete strength in Table 4. Regression analysis results are sufficient in the scope of the current research. The probability of obtaining these results by chance is nearly zero; the significance F value related to variable concrete strength in Table 5 is equal to 9.86 × 10^−6^. In other words, for variable concrete strength, the credibility of correlation results obtained in regression analysis is confirmed by ANOVA analysis.

#### 2.3.2. FRP Reinforcement Ratio

According to the results (as given in Table 4 for variable reinforcement ratio), there exists a positive correlation, equal to 36.1%, between FRP reinforcement ratio and ACI 440.1R-15 discrepancy. Of the variation in theoretical error of nominal flexural moment predicted with the use of the ACI 440.1R-15 design code, 13.0% (value of R Square in Table 4) can be explained by the variation in FRP reinforcement ratio of FRP-reinforced beams. Regression analysis results are sufficient in the scope of the current research. The probability of obtaining these results by chance is nearly zero; Significance F value related to variable reinforcement ratio in Table 5 is equal to 0.00016.

#### 2.3.3. Superposition of Concrete Strength and FRP Reinforcement Ratio

According to the results (as given in Table 4 for variable superposition of concrete strength and FRP reinforcement ratio), there exists a positive correlation equal to 47.6%, between superposition of concrete strength and reinforcement ratio and ACI 440.1R-15 discrepancy. Of the variation in theoretical error of nominal flexural moment predicted with the use of ACI 440.1R-15 design code, 22.6% (value of R Square in Table 4) can be explained by the variation in the superposition of concrete strength and reinforcement ratio. Regression analysis results are sufficient in the scope of current research. The probability of obtaining these results by chance is nearly zero; significance F in Table 5 related to the variable superposition of concrete strength, and FRP reinforcement ratio is equal to 2.08 × 10^−6^.

### 2.4. Correlation Analysis

To identify the character of the correlation between the investigated parameter and error in the theoretical result, correlation analysis was performed at various intervals of data. Correlation between concrete strength and ACI 440.1R-15 discrepancy, correlation between reinforcement ratio and ACI 440.1R-15 discrepancy, and correlation between superposition of concrete strength and reinforcement ratio were investigated at extreme intervals. In other words, the correlation was checked between theoretical error and low strength of concrete, high strength of concrete, low reinforcement ratio, high reinforcement ratio, and also for convergence of high concrete strength and high reinforcement ratio.

In regression and ANOVA analysis, a simple correlation analysis was investigated for all positions related to specific variables in the dataset. Further, more advanced correlation analysis was performed to identify the parts of the dataset for which variation in theoretical error of nominal flexural moment predicted using the ACI 440.1R-15 design code could be explained by the variation in parameter related to the FRP-reinforced beam.

For correlation analysis related to each variable, various datasets were created and analysed. Each dataset consisted of a neutral range of data (all positions from the dataset in Table 2, with theoretical error is not exceeding 10% and where correlation with ACI 440 discrepancy is low) as a reference set, and positions included in the examined extreme interval set.

### 2.5. Nonlinear Generalized Reduced Gradient (GRG) Optimization

Modifications related to results of statistical analysis are proposed to be implemented into the ACI 440.1R-15 analytical model with the use of additional coefficients.

For adjustment and optimization of introduced coefficients, Generalized Reduced Gradient (GRG) analysis was performed. A linear relationship was assumed between coefficients related to two investigated variables and their simultaneous occurrence. The objective of GRG analysis was set to maximum, as an average value of the sum of ACI 440.1R-15 nominal flexural strength of FRP reinforced beam in the current dataset and the proposed coefficient ψ. Average nominal flexural strength predicted with ACI 440.1R-15 model, in the dataset included in the table, was equal to 76.0% of the experimental value, an average theoretical error equal to 0.24. The newly proposed analytical model guarantees average accuracy equal to 93.1%, and improvement in accuracy was observed for 45.7% of specimens.

### 2.6. Implementation of Statistical and Mathematical Results into ACI 440.1R-15 Equations and Proposed Changes in Flexural Design

The influence of the examined factors on nominal flexural strength of concrete beams reinforced with FRP bars was proposed to be implemented to the ACI 440.1R-15 model by the introduction of new coefficients, ψ, (Equation (1)) into design code formula 7.2.2d (Equation (2)). Proposed modification to ACI 440.1R-15 7.2.2d formula is implemented into Equation (3) for compression-controlled section design and into Equation (4) for tension-controlled section design. The reasons for the proposal of such a solution are of practical importance: it guarantees simplicity of flexural design and high accuracy of analytical results obtained.

Values of proposed coefficients (in Equation (1.3)–(1.5)), resulting from GRG analysis, for the transition zone linear relationship are proposed (Equation (1.2)).

**Figure 2:**
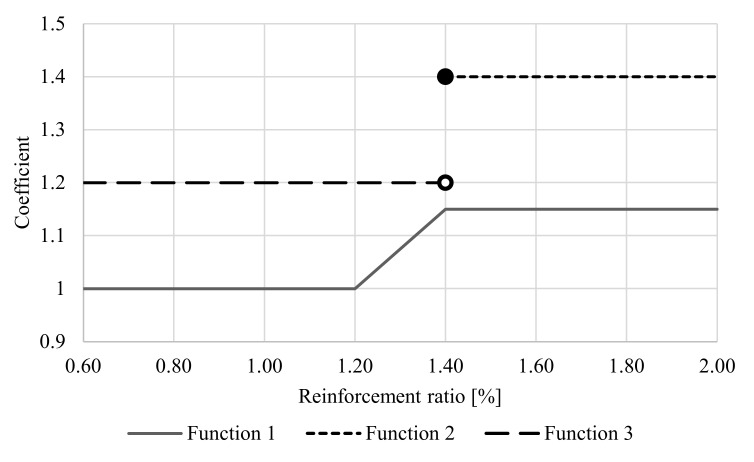
Proposed coefficient as a function of concrete strength and reinforcement ratio.

The proposed coefficient, ψ, is expressed as a function of concrete strength and reinforcement ratio, as shown in Figure 2. Equation (1.1)–(1.3) correspond to Function 1 in Figure 2. Equation (1.4) corresponds to Function 3, and Equation (1.5) corresponds to Function 2.

(1) $$\psi =\left\{\begin{array}{cccc} 1.00 & \mathrm{for} & f_{c}^{’}<55\;\mathrm{MPa}\;\wedge \;\rho_{f}<1.2\% & \left(1.1\right)\\ 0.40+\frac{\rho_{f}}{2} & \mathrm{for} & f_{c}^{’}<55\;\mathrm{MPa}\;\wedge \;1.2\%\%\leq \;\rho_{f}<1.4\% & \left(1.2\right)\\ 1.15 & \mathrm{for} & f_{c}^{’}<55\;\mathrm{MPa}\;\wedge \;\rho_{f}\geq 1.4\% & \left(1.3\right)\\ 1.20 & \mathrm{for} & f_{c}^{’}\geq 55\;\mathrm{MPa}\;\wedge \;\rho_{f}<1.4\% & \left(1.4\right)\\ 1.40 & \mathrm{for} & f_{c}^{’}\geq 55\;\mathrm{MPa}\;\wedge \;\rho_{f}ࣙ1.4\% & \left(1.5\right) \end{array}\right.$$

where:

ψ—proposed coefficient for concrete strength and reinforcement ratio influence; $f_{c}^{’}$—specified compressive strength of concrete, MPa; $\rho_{f}$—FRP reinforcement ratio, %.

∧—the truth-functional operator of logical conjunction; the selected values for the coefficient can be applied if and only if all of the conditions are satisfied.

(2) $$f_{f}=\left(\sqrt{\frac{{\left(E_{f}\times \varepsilon_{cu}\right)}^{2}}{4}+\frac{0.85\times \beta_{1}\times f_{c}^{’}}{\rho_{f}}}\times E_{f}\times \varepsilon_{cu}-0.5\times E_{f}\times \varepsilon_{cu}\right)\leq f_{fu}$$

where:

$f_{f}—$tensile strength of FRP bar in longitudinal direction, MPa;

$f_{fu}—$design tensile strength of FRP bar, MPa;

b—width of rectangular cross-section, mm;

$E_{f}—$elastic modulus of FRP bar in the longitudinal direction, GPa. ;

$\beta_{1}—$safety factor related to concrete strength;

$\varepsilon_{cu}—$ultimate strain in concrete, ‰.

(3) $$f_{f}=\left(\sqrt{\frac{{\left(E_{f}\times \varepsilon_{cu}\right)}^{2}}{4}+\frac{0.85\times \beta_{1}\times \psi \cdot \times }{\frac{\rho_{f}}{\psi }}}\times E_{f}\times \varepsilon_{cu}-0.5\times E_{f}\times \varepsilon_{cu}\right)\leq f_{fu}$$

Since the proposed coefficient is primarily related to the influence of material characteristics on nominal flexural strength of FRP-reinforced beams, it is implemented into the formula for the calculation of tensile strength of FRP bar in a longitudinal direction. In the case of compression-controlled sections, it is effectuated by multiplication of a safety factor related to concrete strength, $\beta_{1}$; by a new coefficient, ψ; and by the division of FRP-reinforced ratio by ψ—a modification of part of Equation (3), which considers material characteristics in the flexural design of FRP reinforced beams. The new coefficient is proposed to be implemented into Equation (4) by multiplication of design tensile strength of FRP bar, $f_{fu}$, with ψ.

(4) $$f_{f}=f_{fu}\times \psi$$

Safety factor related to concrete strength, $\beta_{1}$, provide a flexural bearing capacity reserve for FRP-reinforced beams with concrete strength exceeding 28 MPa. Factor $\beta_{1}$ is taken as 0.85 for concrete strength, $f_{c}^{’}$, up to and including 28 MPa. For concrete strength above 28 MPa, $\beta_{1}$ is reduced continuously at a rate of 0.05 per 7 MPa of strength in excess of 28 MPa but is not taken less than 0.65. Due to factor $\beta_{1},$ a negative influence of increased concrete strength on compressive strength of FRP reinforced beam is considered; tensile strength of FRP bar in longitudinal direction is reduced for FRP reinforced beam with concrete strength lower or equal to 56 MPa. The correctness of this assumption made by ACI 440 is confirmed by data collected in this research (ACI 440 results, Table 6). However, results of this research indicate a positive influence of concrete strength greater than or equal to 55 MPa on nominal flexural strength of concrete beams reinforced with FRP bars.

For these reasons, a modification of safety factor related to concrete strength, $\beta_{1}$, is proposed as follows: factor $\beta_{1}$ is taken as 0.85 for concrete strength, $f_{c}^{’}$, up to and including 28 MPa. For concrete strength above 28 MPa, $\beta_{1}$ is reduced continuously at a rate of 0.05 per 7 MPa of strength in excess of 28 MPa but is not taken less than 0.70. In this case, tensile strength of the FRP bar in the longitudinal direction is reduced in exactly the same manner as before modification for FRP-reinforced beam with concrete strength lower than or equal to 55 MPa. Tensile strength of FRP bar in the longitudinal direction for FRP-reinforced beams with concrete strength greater than or equal to 56 MPa is not further reduced by decreasing safety factor, $\beta_{1}$, to 0.65. Instead, $\beta_{1}$ is kept at a constant value of 0.70 for concrete strength greater than or equal to 49 MPa. A positive influence of concrete strength is considered by multiplication of $\beta_{1}$ by the newly proposed coefficient, ψ, for concrete strength above 55 MPa.

Therefore, the parameter β1 is the factor by which the height of the compression zone of the concrete in bending is determined for the rectangular Whitney stress block. A rectangular stress block is a representation of a nonlinear stress distribution in the concrete. The coefficient β1 = 0.70 was adopted due to the distinction between the compressive strength of concrete of 50 MPa.

## 3. Results and Discussion

To verify the accuracy and safety aspects of the proposed analytical model, the results were compared to experimental results. Table 6 provides theoretical flexural strength calculated using the proposed model and the original ACI 440.1R-15 model. The error of analytical prediction is represented as an absolute value. The proposed model meets safety requirements: all nominal flexural strengths, calculated with the use of the proposed model, of FRP-reinforced beams included in the dataset are underestimated—there exists reserve in their bearing capacity.

The average accuracy of the flexural nominal strength of concrete beams reinforced with FRP bars predicted with the standard ACI 440.1R-15 approach is equal to 87.3%. The newly proposed analytical model guarantees average accuracy equal to 93.1%. An improvement in accuracy is observed for 45.7% of specimens. Moreover, the proposed coefficients were applied in design procedure for beams corresponding to ACI 440 error equal to 7% or more (70.5% of positions with high error); therefore, improvements are significant and observed for most critical situations.

**Figure 3:**
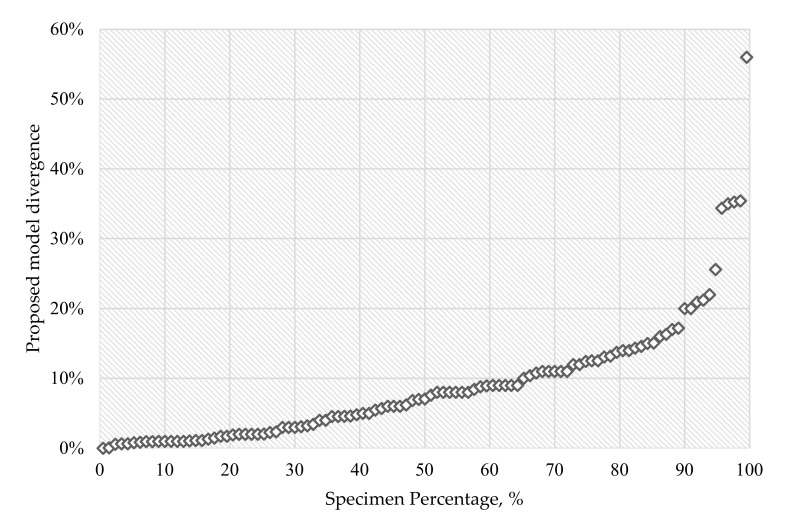
Normal probability plot after implemented changes.

For better visualization of the changes in results after the implementation of the new coefficients, results are marked with colour shades: a green background is equivalent to the most accurate result, and a red background indicates the largest theoretical error. Probability analysis was performed for the dataset containing results obtained with the modified ACI 440.1R-15, and the probability plot for results after implementations of proposed changes (Figure 3) is compared with probability plot for original ACI 440 results (Figure 1).

The normal probability plot of theoretical flexural strength of FRP-reinforced beam error related to the results obtained with the use of the proposed model is nearly linear for up to 89.5% of specimens; the theoretical discrepancy for 91 out of 102 FRP-reinforced beams did not exceed 17.2%. For comparison, the discrepancy of ACI 440 results do not exceed 17.2% for 65.2% of specimens.

The proposed analytical model was not applied to any CFRP-reinforced beam included in Table 2. Statistical analysis on the dataset consisting of CFRP reinforced beams only (data not included in this work due to irrelevance) did not have positive results—no correlation was found in ANOVA analysis. The highest concrete strength used in CFRP-reinforced beams is equal to 53.3 MPa, and the reinforcement ratio does not exceed 1.20; therefore, this dataset is not suitable to be verified in the scope of analytical model with the proposed parameters. An analytical model better suited to concrete beams reinforced with CFRP bars and a higher number of positions in the dataset may be required, as well as, in the case, that of any other type of bars.

## 4. Conclusions

An extensive research programme was realized to investigate the influence of various factors on flexural strength of concrete beams reinforced with FRP bars, as well as the correlation between the error of analytical results of nominal flexural strength of FRP-reinforced beams obtained with the use of ACI 440.1R-15 design code approach and results obtained experimentally. From the statistical and numerical study covered in this work, the following conclusive remarks can be drawn:

1. The nominal flexural strength of FRP reinforced beam predicted with ACI 440 design code matches the experimental results fairly well for beams with low and moderate concrete strength or FRP reinforcement ratio.
2. Theoretical results obtained with the use of ACI 440 design code for beams characterised by high compressive strength or high reinforcement ratio are significantly underestimated.
3. Results of the study in this work indicate a positive influence of interference of application of high-strength concrete and high reinforcement ratio in FRP-reinforced beams on their flexural strength.
4. The proposed coefficient, ψ, is suggested to be implemented into the ACI 440.1R-15 7.2.2d formula for the determination of stress in FRP reinforcement in the tensile zone at ultimate conditions for the compression-controlled section and the tension-controlled section, since the proposed coefficient is a function of factors related to material characteristics.
5. Modifications to the safety factor related to concrete strength, $\beta_{1}$, included in the ACI 440 design approach are proposed in this work due to the positive influence of high and very high concrete strength on the load-bearing capacity of FRP-reinforced beams found in this research.
6. According to regression and ANOVA analysis static scheme, FRP bar type and predicted failure mode does not influence nominal flexural strength of FRP-reinforced beams. However, there may be some restrictions in using these values, as for some other FRP types the values can be not applicable. Authors believe that this method can be generalized, and the values for other types can be defined from other experimental studies and compared with the new model.

Implementation of the proposed coefficient, ψ, into the ACI 440.1R-15 model for flexural design of concrete beams reinforced with FRP bars may provide higher accuracy of theoretical results and thereby assures higher safety and cost efficiency.

## Figures and Tables

**Table 1:** Research programme.

No.	Research Task	Description
1	Data collection	Elaboration of database, supplemented with missing information, required for reliable statistical analysis.
2	Probability analysis	Verification of the viability of the dataset.
3	Regression and analysis of variance (ANOVA)	Investigation of the influence of individual factors on theoretical (ACI 440) differences in the analytical analysis of FRP-reinforced concrete beams. Verification of the significance and usefulness of the obtained results. Interrelation analysis between factors with significant results in superposition.
4	Correlation analysis	Identification of character of correlation between investigated parameter and error in the theoretical result. Identification of the part of the dataset for which variation in the theoretical error of nominal flexural moment predicted with the use of ACI 440.1R-15 design code can be explained by the variation in parameter related to FRP reinforced beam.
5	Nonlinear Generalized Reduced Gradient (GRG) analysis	Adjustment and optimization of introduced coefficients.

**Table 2:** Collected data used for statistical and mathematical analysis.

No.	Ref.	Beam Notation	FRP Type	Failure Mode	Concrete Strength	Reinforcement Ratio	Load Bearing		
							Test	ACI	Test/ACI
					MPa	%	kNm	kNm	%
1	[16]	COMP-00	GFRP	Concrete	35.40	1.33	40.3	37.0	0.92
2	[16]	COMP-25	GFRP	Concrete	36.40	1.33	40.3	37.4	0.93
3	[16]	COMP-8O	GFRP	Concrete	36.50	1.33	40.3	37.4	0.93
4	[16]	COMP-75	GFRP	Concrete	37.50	1.33	44.3	38.1	0.86
5	[13]	BC2HA	GFRP	Concrete	57.20	1.16	19.7	15.8	0.80
6	[13]	BC2HB	GFRP	Concrete	57.20	1.16	20.6	15.9	**0.77**
7	[13]	BC2VA	GFRP	Concrete	97.40	1.16	22.7	15.7	**0.69**
8	[13]	BC4NB	GFRP	Concrete	46.20	2.70	20.6	16.9	0.82
9	[13]	BC4HA	GFRP	Concrete	53.90	2.70	21.0	17.9	0.85
10	[13]	BC4HB	GFRP	Concrete	53.90	2.70	21.4	17.8	0.83
11	[13]	BC4VA	GFRP	Concrete	93.50	2.70	28.4	18.5	**0.65**
12	[13]	BC4VB	GFRP	Concrete	93.50	2.70	29.5	18.3	**0.62**
13	[14]	C1-4	CFRP	Concrete	40.40	0.60	71.2	59.1	0.83
14	[14]	C1-6	CFRP	Concrete	39.30	0.90	83.1	74.0	0.89
15	[14]	C1-8	CFRP	Concrete	39.30	1.20	90.4	82.3	0.91
16	[14]	C2-4	CFRP	Concrete	39.90	0.50	78.8	63.0	0.80
17	[14]	C2-6	CFRP	Concrete	40.80	0.80	80.9	74.4	0.92
18	[14]	C2-8	CFRP	Concrete	40.80	1.10	89.4	82.2	0.92
19	[14]	G1-6	GFRP	Concrete	39.05	1.60	77.5	62.8	0.81
20	[14]	G-8	GFRP	Concrete	39.05	2.20	86.8	69.4	0.80
21	[14]	G2-6	GFRP	Concrete	39.05	1.40	71.0	56.8	0.80
22	[14]	G2-8	GFRP	Concrete	39.05	1.90	84.5	63.4	**0.75**
23	[15]	Beam2	GFRP	FRP	27.68	0.23	5.9	5.7	0.97
24	[15]	Beam4	GFRP	FRP	27.68	0.17	7.8	7.5	0.95
25	[15]	Beam6	GFRP	FRP	27.68	0.14	10.8	9.2	0.85
26	[15]	Beam8	GFRP	FRP	50.09	0.23	5.9	5.8	0.99
27	[15]	Beam10	GFRP	FRP	50.09	0.17	9.5	7.6	0.80
28	[15]	Beam12	GFRP	FRP	50.09	0.14	16.8	18.6	**1.11**
29	[11]	1FRP1	GFRP	FRP	27.60	0.12	11.5	11.3	0.98
30	[11]	1FRP2	GFRP	FRP	27.60	0.12	12.7	11.3	0.89
31	[11]	1FRP3	GFRP	FRP	27.60	0.12	11.5	11.3	0.98
32	[11]	2FRP1	GFRP	FRP	27.60	0.13	13.6	12.0	0.88
33	[11]	2FRP2	GFRP	FRP	27.60	0.13	13.3	12.1	0.91
34	[11]	2FRP3	GFRP	FRP	27.60	0.13	13.1	12.0	0.92
35	[11]	4FRP1	GFRP	Concrete	27.60	1.27	15.8	13.6	0.86
36	[11]	4FRP2	GFRP	Concrete	27.60	1.27	15.6	13.7	0.88
37	[11]	4FRP3	GFRP	Concrete	27.60	1.27	16.3	13.7	0.84
38	[11]	5FRP1	GFRP	Concrete	27.60	1.35	16.4	13.1	0.80
39	[11]	5FRP2	GFRP	Concrete	27.60	1.35	16.7	13.2	**0.79**
40	[11]	5FRP3	GFRP	Concrete	27.60	1.35	15.8	13.1	0.83
41	[17]	CB2B-1	GFRP	Concrete	52.00	0.69	57.9	53.8	0.93
42	[17]	CB2B-2	GFRP	Concrete	52.00	0.69	59.8	53.8	0.90
43	[17]	CB3B-1	GFRP	Concrete	52.00	1.04	66.0	64.0	0.97
44	[17]	CB3B-2	GFRP	Concrete	52.00	1.04	64.8	64.2	0.99
45	[17]	CB4B-1	GFRP	Concrete	45.00	1.47	75.4	62.6	0.83
46	[17]	CB4B-2	GFRP	Concrete	45.00	1.47	71.7	62.4	0.87
47	[17]	CB6B-1	GFRP	Concrete	45.00	2.20	84.8	72.9	0.86
48	[17]	CB6B-2	GFRP	Concrete	45.00	2.20	85.4	73.4	0.86
49	[18]	1	GFRP	FRP	35.90	0.38	7.0	7.0	0.99
50	[18]	2	GFRP	FRP	36.90	0.38	6.6	7.0	**1.06**
51	[18]	4	GFRP	FRP	38.90	0.38	7.2	7.2	1.00
52	[18]	5	GFRP	FRP	39.90	0.38	7.4	7.3	0.99
53	[18]	6	GFRP	FRP	40.90	0.38	6.8	7.4	**1.09**
54	[19]	GB10	GFRP	Concrete	39.80	1.36	39.5	36.3	0.92
55	[19]	GB9	GFRP	Concrete	39.80	1.36	39.7	36.2	0.91
56	[19]	GB5	GFRP	Concrete	31.20	1.36	40.3	32.6	0.81
57	[12]	RC2	GFRP	Concrete	30.00	0.70	36.8	37.9	**1.03**
58	[12]	RC4	GFRP	Concrete	30.00	1.22	60.7	47.3	**0.78**
59	[20]	C-212-D1	GFRP	Concrete	59.80	0.99	38.2	23.7	**0.62**
60	[20]	C-216-D1	GFRP	Concrete	56.30	1.78	45.1	28.8	**0.64**
61	[20]	C-316-D1	GFRP	Concrete	55.20	2.67	49.4	33.1	**0.67**
62	[20]	C-212-D2	GFRP	Concrete	39.60	0.99	27.7	18.0	**0.65**
63	[20]	C-216-D2	GFRP	Concrete	61.70	1.78	42.2	25.7	**0.61**
64	[20]	C-316-D2	GFRP	Concrete	60.10	2.67	43.2	29.4	**0.68**
65	[21]	C-S-1	CFRP	FRP	26.90	0.42	64.1	53.9	0.84
66	[21]	C-S-2	CFRP	FRP	27.50	0.16	44.3	42.1	0.95
67	[21]	C-C-3	CFRP	FRP	23.60	0.16	44.8	42.1	0.94
68	[21]	C-C-4	CFRP	FRP	27.20	0.42	60.7	54.0	0.89
69	[21]	C-C-5	CFRP	FRP	28.00	0.42	56.0	53.8	0.96
70	[22]	CS1a	CFRP	Concrete	26.00	0.42	51.8	52.8	**1.02**
71	[22]	CS1b	CFRP	Concrete	26.00	0.28	29.0	45.2	**1.56**
72	[22]	GS1a	GFRP	Concrete	28.00	1.18	60.2	56.6	0.94
73	[22]	GS1b	GFRP	Concrete	28.00	0.79	49.0	48.0	0.98
74	[23]	B4	CFRP	Concrete	51.73	0.34	12.6	12.7	1.01
75	[23]	B5	CFRP	FRP	48.02	0.34	10.2	12.4	**1.22**
76	[23]	B7	CFRP	Concrete	49.30	0.53	17.1	14.7	0.86
77	[23]	B8	CFRP	FRP	51.10	0.53	16.9	14.9	0.88
78	[23]	B12	CFRP	FRP	43.88	0.76	17.5	16.1	0.92
79	[23]	B9	CFRP	FRP	53.31	0.53	16.6	15.1	0.91
80	[24]	80-#2-0.5	GFRP	FRP	95.00	0.50	15.0	11.4	**0.76**
81	[24]	80-#3-1.0	GFRP	Concrete	95.00	1.00	33.0	27.1	0.82
82	[24]	80-#4-2.0	GFRP	Concrete	95.00	2.00	46.1	32.3	**0.70**
83	[24]	120-#2-0.5	GFRP	FRP	117.00	0.50	16.2	11.3	**0.70**
84	[24]	120-#3-1.0	GFRP	Concrete	117.00	1.00	41.8	30.5	**0.73**
85	[24]	120-#4-2.0	GFRP	Concrete	117.00	2.00	52.2	36.0	**0.69**
86	[25]	CFRRP1 (2)	CFRP	Concrete	40.40	0.60	74.6	76.9	**1.03**
87	[25]	CFRRP1 (3)	CFRP	Concrete	39.30	0.90	83.1	79.3	0.95
88	[25]	CFRRP1 (4)	CFRP	Concrete	39.30	1.20	90.4	87.5	0.97
89	[25]	CFRP 2 (1)	CFRP	Concrete	39.90	0.50	78.8	67.5	0.86
90	[25]	CFRP 2 (2)	CFRP	Concrete	39.90	0.50	78.2	74.6	0.95
91	[25]	CFRP 2 (3)	CFRP	Concrete	40.80	0.80	80.9	80.2	0.99
92	[25]	CFRP 2 (4)	CFRP	Concrete	40.80	1.10	89.4	88.7	0.99
93	[25]	GFRP1 (1)	GFRP	Concrete	39.05	1.60	77.5	67.0	0.86
94	[25]	GFRP1 (2)	GFRP	Concrete	39.05	2.20	86.8	74.5	0.86
95	[25]	GFRP2 (1)	GFRP	Concrete	39.05	1.40	71.0	61.1	0.86
96	[25]	GFRP2 (2)	GFRP	Concrete	39.05	1.90	84.5	68.3	0.81
97	[26]	FB20	CFRP	Concrete	25.20	0.40	84.8	82.8	0.98
98	[26]	FB19	CFRP	Concrete	29.00	0.30	105.6	68.2	**0.65**
99	[26]	FB18	CFRP	Concrete	29.80	0.21	64.0	50.6	**0.79**
100	[26]	FB17	CFRP	Concrete	22.30	0.40	84.0	79.4	0.95
101	[26]	FB16	CFRP	Concrete	25.10	0.30	102.4	66.3	**0.65**
102	[26]	FB15	CFRP	Concrete	21.30	0.21	73.6	48.3	**0.66**

**Table 3:** Summary of ANOVA analysis.

Variable	Factor	Significance × 10^−6^	Correlation	Implementation into Analytical Model
1	Static scheme	433,685.00	0.07721	No. Results NOT significant
2	FRP bar type	188,500.00	0.12934	No. Results NOT significant
3	Concrete strength	9.86	**0.41657**	Yes. Results significant
4	Reinforcement ratio	156.00	**0.36082**	Yes. Results significant
5	Failure mode	2455.00	0.29259	No. Results NOT significant
6	Year of research	14,208.00	0.23868	No. Results NOT significant
7	3 + 4 variables	2.08	**0.47566**	Yes. Results significant
8	4 + 5 variables	471,000.00	0.37344	No. Results NOT significant

**Table 4:** Results of regression analysis.

Regression Statistics	Concrete Strength	Reinforcement Ratio	Concrete Strength + Reinforcement Ratio
Multiple R	0.41657	0.36082	0.47566
R Square	0.17353	0.13019	0.22626
Adjusted R Square	0.16551	0.12175	0.21109
Standard Error	0.10234	0.10499	0.09951
Observations	102	102	102

**Table 5:** Results of ANOVA analysis.

Variable	ANOVA	df	SS	MS	F	Significance F
Concrete strength	Regression	1	0.226528	0.226528	21.627121	9.86 × 10^−6^
	Residual	103	1.078847	0.010474		
	Total	104	1.305375			
Reinforcement ratio	Regression	1	0.169949	0.169949	15.416943	0.000156
	Residual	103	1.135426	0.011024		
	Total	104	1.305375			
Concrete strength and Reinforcement ratio	Regression	2	0.295351	0.147675	14.913373	2.08 × 10^−6^
	Residual	102	1.010025	0.009902		
	Total	104	1.305375			

**Table 6:** Comparison between ACI 440 results and results obtained with the use of the proposed model (The background color shows the level of disperience).

No.	ψ	Proposed Model Result	ACI 440 Result	Proposed Model Error	ACI 440 Disperience
43	1.00	1.00	0.87	0.00	0.13
93	1.08	0.99	0.86	0.01	0.14
53	1.08	0.99	0.92	0.01	0.08
54	1.00	0.99	0.92	0.01	0.08
2	1.07	0.99	0.93	0.01	0.07
3	1.00	0.99	0.93	0.01	0.07
95	1.15	0.99	0.86	0.01	0.14
44	1.15	0.99	0.86	0.01	0.14
45	1.15	0.99	0.86	0.01	0.14
94	1.20	0.99	0.86	0.01	0.14
81	1.08	0.99	0.82	0.01	0.18
52	1.08	0.98	0.91	0.02	0.09
55	1.40	0.98	0.91	0.02	0.09
82	1.00	0.98	0.70	0.02	0.30
1	1.15	0.98	0.92	0.02	0.08
9	1.00	0.98	0.85	0.02	0.15
85	1.00	0.97	0.69	0.03	0.31
5	1.15	0.96	0.80	0.04	0.20
42	1.15	0.95	0.83	0.05	0.17
10	1.00	0.95	0.83	0.05	0.17
64	1.00	0.95	0.68	0.05	0.32
8	1.00	0.94	0.82	0.06	0.18
61	1.15	0.94	0.67	0.06	0.33
19	1.00	0.93	0.81	0.07	0.19
96	1.20	0.93	0.81	0.07	0.19
6	1.00	0.92	0.77	0.08	0.23
21	1.15	0.92	0.80	0.08	0.20
20	1.07	0.92	0.80	0.08	0.20
4	1.20	0.92	0.86	0.08	0.14
80	1.04	0.91	0.76	0.09	0.24
36	1.00	0.91	0.88	0.09	0.12
11	1.00	0.91	0.65	0.09	0.35
60	1.08	0.90	0.64	0.10	0.36
40	1.04	0.89	0.83	0.11	0.17
33	1.00	0.89	0.86	0.11	0.14
84	1.08	0.88	0.73	0.12	0.27
51	1.08	0.87	0.81	0.13	0.19
56	1.04	0.87	0.81	0.13	0.19
37	1.40	0.87	0.84	0.13	0.16
12	1.15	0.87	0.62	0.13	0.38
22	1.08	0.86	0.75	0.14	0.25
35	1.00	0.86	0.80	0.14	0.20
63	1.00	0.85	0.61	0.15	0.39
39	1.00	0.85	0.79	0.15	0.21
83	1.00	0.84	0.70	0.16	0.30
7	1.00	0.83	0.69	0.17	0.31
58	1.20	0.79	0.78	0.21	0.22
59	1.00	0.74	0.62	0.26	0.38

## Data Availability

Data sharing is not applicable to this article.
